# Observations upon Military Hospitals and Army Medical Practice

**Published:** 1862-10

**Authors:** 


### EDITORIAL DEPARTMENT.

### OBSERVATIONS UPON MILITARY HOSPITALS AND ARMY MEDICAL PRACTICE.

By appointment from the Mayor of Buffalo and the Senatorial District War Committee, Prof. Sandford Eastman and myself have had confided to us the duty of visiting the wounded soldiers of the 21st Regiment N. Y. V., thus affording us the opportunity of visiting the Army, and Hospitals in Washington, Georgetown and Alexandria, and observing the general condition and practice as adopted in these institutions.

In our mission, we were received by surgeons, and assistant surgeons with a cordiality and politeness which shows the good breeding and gentlemanly manners of the medical profession as a class, and which is so inwrought into their habits of life and manner of thought, as to be undimmed by place, and unchanged by "tape" or "lace." As a body the army practitioners, so far as observed, are noble souls, intelligent, active, untiring, generous and loyal, caring for the wounded and the sick, with a gentleness and ability pleasant to observe, and which formed a relief in one of the saddest and most sorrowful scenes ever gazed upon by human eyes. Not that the sick and the wounded are alone the objects of deepest sympathy; mothers seeking the bed-side of a son, or watching by the dying couch; fathers and brothers inquiring for the wounded and missing, or weeping at the brief history of "wounded and left on the field," these, and such like scenes form, if not the ground work, at least the shadings of a picture which human language cannot paint.

As is well known, the Capitol has been converted into a hospital. The beds consist of iron bedstead with folding head and foot, good slats supporting a straw or grass mattrass, sheets and white cover, making a neat and comfortable bed. These are placed in the Senate Chamber, Hall of Representatives, and through the long, high, well ventilated halls and apartments of the building generally. Some of the floors of this hospital are of the most elaborate Mosaic work, while the walls are ornamented with paintings by the most distinguished artists, and sculpture upon which genius has drawn its finest lines. Everything which National wealth and taste could impart has been expended upon this institution until we do not hesitate to affirm that no where upon this round globe was there ever built so magnificent and princely a hospital.

The other hospitals in Washington require a notice, for though they are built in very different style, and at comparatively *small cost*, yet to physicians they possess an equal interest, indeed also to the army an equal value. It is but the work of a few hours to build a hospital which will accommodate several acres of wounded men, equally well with the Capitol, upon which centuries of labor and millions of money have been expended.

To organize a military hospital, the country residence of some wealthy gentleman is first appropriated; this is used for "*Head Quarters*." Every thing in the army must have a "head quarters," and a soldier carrying a musket must walk back and forth, before the door. Since the soldier gave us no trouble or rendered us any assistance, we think perhaps the object is simply to *tend the musket*. "Head Quarters" consists of Surgeon's Office, Apothecary Shop or Hospital Steward's Office, General Office where the clerks keep, or pretend to keep, the register, and various other apartments for the comfort of the attendants generally. Outside are either pavilion tents, or temporary sheds, from twenty feet wide by from thirty to one hundred feet long, and these are numbered or lettered, and called wards, the larger hospitals containing from fifty to one hundred such wards. The ventilation is easy, natural, and usually complete.

The wards are often rather uncomfortably near, and though *out door, is convenient and plenty*, it is not always so agreeable. Direct ventilation is, after all, the cheapest and best. A tent with the sides and ends open, in the warm season, is really an exceedingly good place for cases of fever or any other. The temporary wood buildings are also well aired, though not quite so fully as the tents, and when placed side by side, in great numbers, it requires some effort to get free and perfect ventilation. These larger hospitals occupy often times from ten to thirty acres of land, and accommodate as many patients as circumstances require. Government I think has about thirty hospitals organized in Washington, some of which are small, containing perhaps not more than one hundred beds, and occupy churches or other large buildings for all purposes; but for the most part they are immensely large, and the physician accustomed only to the wards of our civil hospitals, is struck with amazement that the acres of wounded and sick before him should be said to be in a hospital. The bedding is the same in character as that in the National Palace, clean, white, and uniform. Physicians are attentive, kind and indulgent in a most marked and noticable degree, thus also ensuring the same conduct from others under their direction. In hospitals and wards supplied with female nurses, there is very decided superiority in the cleanliness and general air of comfort, and no one can fail to observe the beneficial influence they exert, to say nothing of the superiority of their services in many departments. The hospitals in Georgetown and Alexandria are of the same general description, though I think it cannot be truthfully said that in Alexandria they are as well arranged, cleanly and comfortable, as in Washington. Civilians visit these hospitals going from the pure air into the crowded wards and returning again think them offensive in odor, and disgusting in the extreme. This grows out of want of experience in these matters, and a feeling that a hospital ward should be as clear and pure in its atmosphere as a private dwelling. We have heard exaggerated accounts given and very untruthful statements made as to the condition of these apartments. Of all the abuses practiced in the army, in the hospitals there is the least of which to complain.

Practice as observed in these hospitals does not consist in surgical operations, as many are led to suppose. A great mistake would have been avoided had military practitioners been called physicians; a mistake which has had some influence in determining the character of the Army Medical Staff. The great want in this department is experienced physicians, not expert or distinguished surgeons. By this we do not desire to be understood that any deficiency in medical ability was observed in those now practicing, but that the name of surgeon has kept from the army some of the most experienced and capable physicians, who would have been signally useful either in the care of regiments in the field or of military hospitals.

The great mass of wounds require no operative treatment. A good nurse when once instructed how to dress a simple gun-shot wound, can proceed through the ward with little assistance and properly attend to at least three-fourths of the inmates. Indeed the attention required in gun-shot wounds, uncomplicated by injuries of the bones, is exceedingly limited and simple. Cleanliness, tonics, stimulants, abundant and nutritious food will comprise the staples for the successful management of this class of injuries; and this class fortunately will be found to include an astonishingly large proportion of the cases admitted to hospital.

All gun-shot injuries of bone are serious wounds, involving a risk of the part, and if of the larger bones, of life. Bone which has been violently struck by a ball, is not only fractured often times in various directions, but its vitality is destroyed for some distance; it is blackened and necrosed, affected in something the same way as the soft parts, after violent contusion. Fracture by musket ball requires a long time for repair, and in many cases exfoliation of large quantities of dead bone must take place before the processes of union can commence. Fracture of the larger bones will prove fatal in great proportion, but we must wait for statistics to show the fatal numbers. In fracture of the femur, when primary amputation has been neglected, the choice remains of death after secondary operation, or without it; it would be difficult, in many cases to decide, in which direction would be found the fewest chances. In all this vast army of invalids, comparatively few trophies of operative surgery are now seen; and the same may be said of conservative surgery, so far as fracture is concerned, but few, very few cases, in any degree advanced in recovery, can now be observed, though recent cases are sufficiently common.

Fractures of the leg and thigh are dressed generally either upon Smith's Anterior Splint, or laid upon a mattress, and retained in position by sand-bags. With few exceptions fractures are dressed without extension and opportunity will thus be afforded of estimating its advantages provided extension is applied in sufficient frequency to allow comparison. Whatever may be the amount of shortening in these cases, it must not be attributed to neglect of extension; from the nature of the injury to the bone, shortening must necessarily be much greater than usual in fracture from falls, blows, &c. At first we were delighted to think what conclusive evidence we should have of the advantages or disadvantages of extension in treatment of fracture; but a little more observation and reflection convinced us that these results, whatever they might be, would prove nothing of its value or worthlessness in civil practice. Most surgeons, perhaps, regard its necessity as established, and attribute shortening to its insufficiency; however this may be, it is not so conclusively settled as to make further observation useless in estimating the differences in results obtained from its use, or neglect.

Almost all the wounded admitted to hospital receive their injuries from the fire of musketry; two reasons for which may be assigned, viz: artillery fire does not wound nearly as many, and is much more fatal. It is very surprising how musket balls will pass through important and often apparently vital parts, and yet recovery take place; the escape of important vessels in some cases is almost miraculous.

Soldiers cannot estimate in any degree their chances of recovery; the simplest wounds appear to them of equal importance with the severer.—The ball leaves about the same external evidences of injury, whatever may be the internal destruction, and gun-shot wound of thigh with fracture of the femur, is often looked upon as favorably as if uncomplicated by injury of the bone. This is perhaps a great advantage since hope is not lost, and confident expectation of recovery is generally indulged. Young soldiers are very generally hopeful and confident, ready again to test their bravery and patriotism by the fearful chances of battle.

Military practice must be exceedingly tiresome, monotonous and often unpleasant, though its wild life of adventure possesses an attraction which reconciles the military practitioner to his duty. It is certainly a life of hardship and privation, requiring great physical strength to endure, and besides is not altogether free from the common dangers of war. We were told that army dictation had found way also into the medical department, and that in place of advice which has usually been sufficient to produce change of treatment, when kindly and frankly given, a medical director sends to the physician in charge of a hospital or ward, an *order* for removal of water dressing in given case, and application of poultice, or an *order* to amputate immediately an arm or leg. This order must be immediately obeyed or the physician is *ordered* to report to "head quarters," and answer to the charge of disobedience or neglect. Everything in the army it appears must be done by order, individual opinion altogether ignored. It may be that this is necessary, and on the whole best, but it looks to us as altogether unsuited and unbecoming the dignity of the profession, and certain to weaken and destroy individual interest, effort and responsibility, and breed disgust and contempt for those who practice it.

Abuses and mistakes in the medical, as in all other departments of the army, are no doubt sufficiently common, not only in its general direction, but in the details of every day practice. It is not to be supposed that this immense system of provision for the sick and wounded could be conducted without some errors which time and experience only can correct.

Other topics which we should be glad to introduce to the notice of our readers, we must defer for want of time and space.

